# The Buffalo Medical College—Resignation of Prof. E. V. Stoddard

**Published:** 1881-05

**Authors:** 


### THE BUFFALO MEDICAL COLLEGE. RESIGNATION OF PROFESSOR E. V. STODDARD.

The resignation of Dr. E. V. Stoddard, of the Chair of Materia Medica and Hygiene, which he has held for eight years in the University of Buffalo, will be received by the Alumni and friends of this institution with great surprise and regret. Dr. Stoddard has filled this position with rare ability and increasing acceptableness, his last course of lectures having met with the warm endorsement of the class, which found expression in an unanimous request for their publication. We learn that considerations connected with his large and growing practice in Rochester, and the interference with its requirements on account of the necessary absence from his post, in order to deliver his lectures in this city, have induced him to relinquish the position and to sever his connection with the college. The uniformly cordial relations sustained by him, from the first, with the faculty and the profession in this city, make the change a matter of deep regret to all.

We are permitted to announce that for the session of 1881–82, the course of lectures on Materia Medica will be given by Dr. A. R. Davidson, and that on Hygiene by Dr. Thos. Lothrop. Our editorial colleagues, who are thus honored with additional labors and responsibilities, will find in the positions they are invited to fill, a wide and, we hope, a congenial field for the exercise of their professional ability and attainments.

